# Strong light coupling effect for a glancing-deposited silver nanorod array in the Kretschmann configuration

**DOI:** 10.1186/1556-276X-9-567

**Published:** 2014-10-13

**Authors:** Yi-Jun Jen, Wei-Chih Liu, Jung-Hui Chao, Jyong-Wei Huang, Yuan-Tai Chang

**Affiliations:** 1 Department of Electro-Optical Engineering, National Taipei University of Technology No. 1, Sec. 3, Chung-Hsiao E. Rd, Taipei 106, Taiwan

**Keywords:** Glancing angle deposition, Nanorod array, Attenuated total reflection, Light extinction

## Abstract

In this work, three slanted silver nanorod arrays (NRAs) with different thicknesses are fabricated using the glancing angle deposition method. Each silver NRA in the Kretschmann configuration is arranged to form a prism/NRA/air system. Attenuated total reflection occurs over the visible wavelengths and wide incident angles of both s- and p-polarization states. The extinctance is inversely proportional to the thickness of the Ag NRA. The thinnest NRA, with a thickness of 169 nm, exhibits strong extinctance of more than 80% over the visible wavelengths. The associated forward scatterings from the three NRAs are measured and compared under illumination with a laser beam with a wavelength of 632.8 nm.

## Background

Reflection, transmission, absorption, and scattering are the four optical responses of a material that is illuminated with an electromagnetic wave. The development of optical thin films and nanotechnology enables the manipulation of reflection and transmission at designated wavelengths and angles of incidence [1,2]. Light is scattered from a micro- or nanostructure whose characteristic size is near or above the wavelength of illuminated light. A nanoparticle array typically converts most of the input power into reflection, transmission, and absorption and only a small part into the scattering energy [3]. For a metal nanostructure, some of the energy of incident light is used in extinction, including absorption and scattering, and some is used in transmission and reflection. Recently, optical scattering from metallic nanoparticles has been applied in bio-sensing, such as in surface-enhanced Raman scattering (SERS) [4,5] and surface-enhanced fluorescence [6,7]. Many efforts have been made to strengthen the local electric field via local surface plasmonic resonance. However, coupling light energy into the nanoparticles is important to increase scattering by diminishing both reflection and transmission. It is usually difficult to raise light coupling as the incident angle exceeds the Brewster angle because the reflection increases with the incident angle for both p- and s-polarization states. When the incident angle exceeds the critical angle, the total reflection effect would further reduce the light coupling effect. In this work, a slanted silver nanorod array (NRA) is arranged in the Kretschmann configuration to couple incident light energy into the Ag NRA by eliminating reflection and transmission. As the angle of incidence increases over the critical angle of the prism/NRA/air system, diminished s-polarized and p-polarized specular reflectances increase the efficiency of the coupling of the light energy into the Ag NRA. In a previous study, gold particles were arranged in an evanescent field that was generated by the total internal reflection of light from a halogen lamp in a glass prism. The light that was scattered by individual particles was collected using a conventional microscope and spectrally analyzed by a nitrogen-cooled charge-coupled device array that was coupled to a spectrometer [8]. However, the magnitude of the coupling of incident power into the nanoparticle array remains unknown and must be enhanced.

The NRA of interest herein is a silver NRA. Deposited silver NRAs have been fabricated, measured, and analyzed for the past 10 years [9]. Tilting the substrate during deposition causes a columnar array to grow owing to the self-shadowing effect. When the tilted Ag NRA is illuminated by electromagnetic waves, anisotropic absorption is observed because the oscillation of the electric field along and perpendicular to the rod induces the longitudinal plasmon mode (LPM) and the transverse plasmon mode (TPM) [10]. The deposition plane is defined by the direction of growth of the rods and the surface normal. An electric field that oscillates parallel (vertical) to the deposition plane is defined as p(s)-polarized. For a light wave that is normally incident upon a silver NRA, the reflection and absorption of the p-polarized light is higher than those of the s-polarized light for the optical wavelengths that excite the LPM. Our recent work revealed that the permeability of a silver NRA departs from unity, and the real part of the refractive index for p-polarized light was measured to be negative for a glancing-deposited silver NRA with a thickness of 160 nm [9].

When the impedance and refractive index were considered independently of each other, the optical responses of a metal NRA are more diverse than those of a traditional optical thin film whose impedance and refractive indices are the reciprocals of each other. In this work, three glancing-deposited silver NRAs with thicknesses of 169, 198, and 269 nm are arranged in the Kretschmann configuration in the prism coupling system BK7 prism/NRA/air to observe the optical coupling effect. Reflectance is measured versus wavelength and incident angle under total reflection. The measurements indicate that the extinction in the system is enhanced under the conditions of total reflection for visible wavelengths over a large range of angles. The measured extinctance spectra indicate that extinctance is inversely proportional to the thickness of the NRA. The associated forward scattering into air is measured and compared with that of NRA under a normal incidence of a laser beam with a wavelength of 632.8 nm upon the air/NRA/glass system.

## Methods

**Figure 1:**
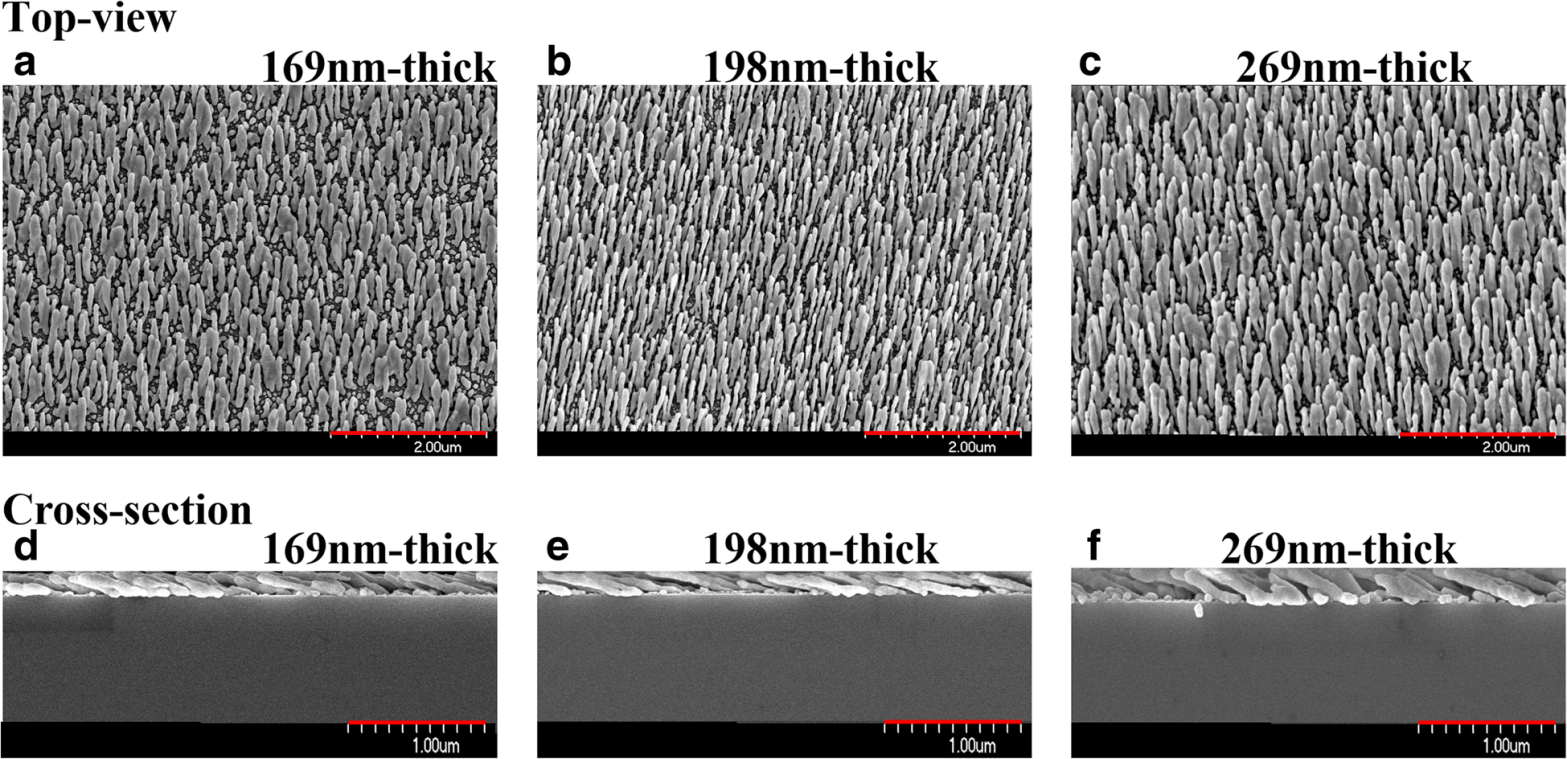
**Top view and cross-section SEM images. (a, d)** Ag NRA with a thickness of 169 nm. **(b, e)** Ag NRA with a thickness of 198 nm. **(c, f)** Ag NRA with a thickness of 269 nm

Electron beam evaporation [11,12] was applied to grow Ag NRAs. In this work, the substrate normal was tilted at an angle of 89° from the direction of incidence of the vapor during the deposition process. The center of the substrate and the evaporation source separated vertically by a distance of 290 mm. The deposition rate of Ag is maintained at 3 Å/s. Pumping yields a background pressure of 4 × 10^−6^ torr before evaporation. Ag NRAs with thicknesses of 169, 198, and 269 nm are thus fabricated. Figure 1 presents the top view and cross-section scanning electron microscopic (SEM) images of Ag NRAs with thicknesses of 169, 198, and 269 nm, respectively. The average diameter of the nanorods of the three samples is around 80 nm. The angle between the normal direction of the substrate surface and the average orientation of growth of the rods is about 66°.

## Results and discussion

**Figure 2:**
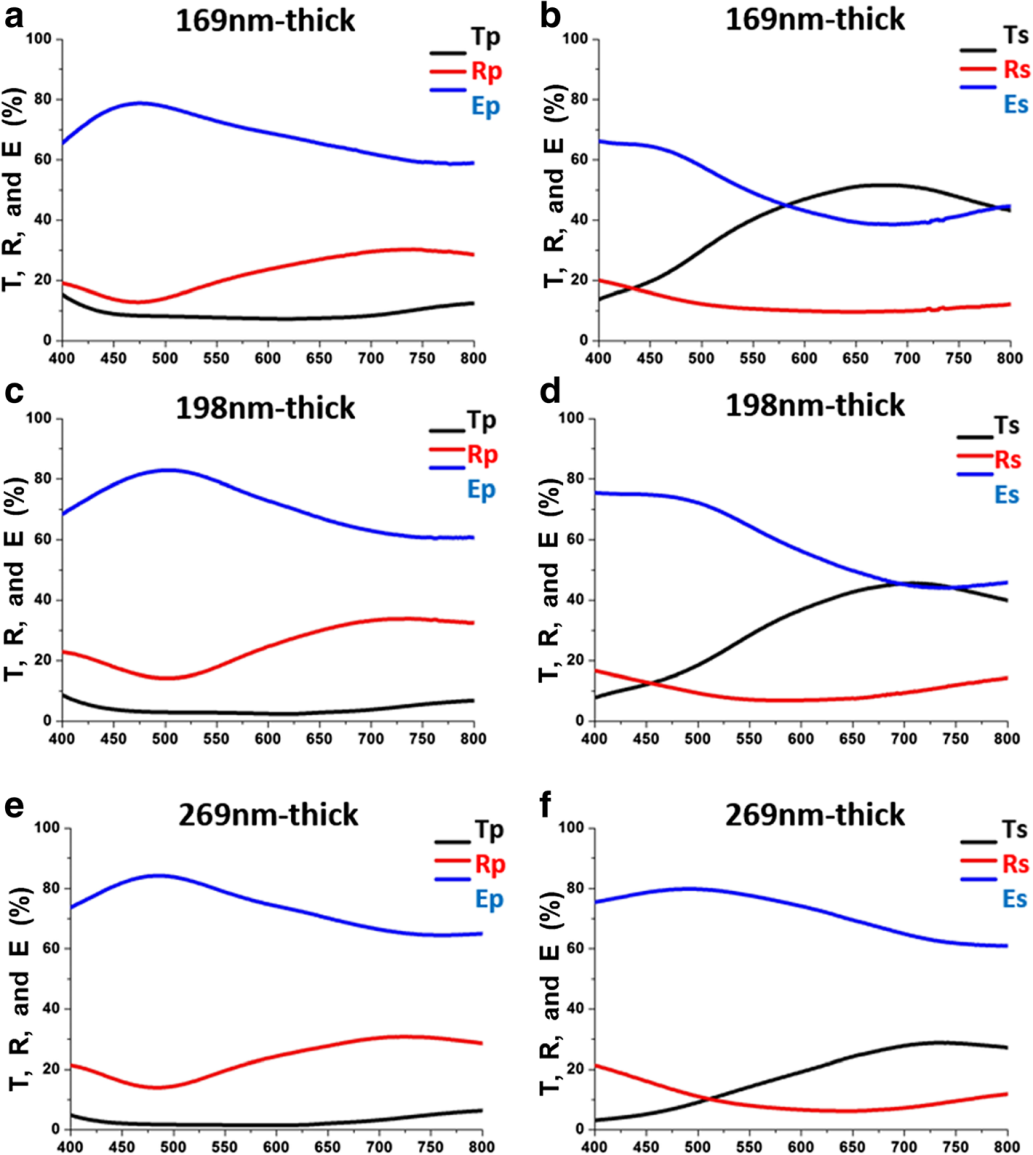
**Transmittance and reflectance spectra measured at normal incidence.** Measured Ag spectra of transmittance *T*_*j*_, reflectance *R*_*j*_, and extinctance *E*_*j*_, *j* ∈ {*p*, *s*}, of NRA with thickness of **(a, b)** 169 nm, **(c, d)** 198 nm, and **(e, f)** 269 nm.

The transmittance and reflectance spectra are measured at normal incidence, as shown in Figure 2. The s-polarized and p-polarized transmittance and reflectance spectra are similar to those obtained elsewhere [9]. For 169-nm-thick Ag NRA, an average *T*_*p*_ of around 9.6% is obtained in the visible regime, and *T*_*p*_ rises from 13.7% at 400 nm to 51.3% at 700 nm. For the 198-nm-thick Ag NRA, the average *T*_*p*_ is approximately 3.7% in the visible regime, and *T*_*s*_ rises from 7.7% at 400 nm to 45.2% at 700 nm. For the 269-nm-thick Ag NRA, the average *T*_*p*_ is approximately 2.6% in the visible regime, and *T*_*s*_ increases from 3.1% at 400 nm to 27.7% at 700 nm.

**Figure 3:**
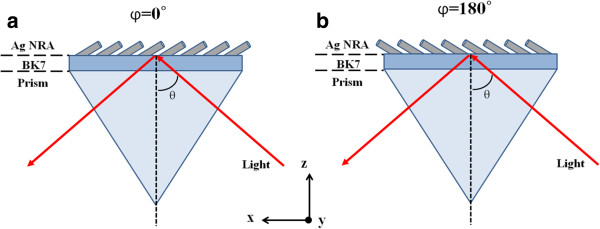
**NRA nanostructure in BK7 glass prism/NRA/air.** Deposition plane, defined by directions of rod and surface normal, is orientated at angles of **(a)***φ* = 0° and **(b)***φ* = 180°. The angle of incidence *θ* and the azimuthal angle *φ* are indicated.

The Ag NRA was arranged in the Kretschmann configuration as a BK7 prism/Ag NRA/air system with its deposition plane coincident with the plane of incidence, as shown in Figure 3. The angle between the deposition plane and the plane of incidence is *φ* = 0° or *φ* = 180° associated with two possible directions of the rods. Reflectance was measured at angles of incidence from *θ* = 45° to *θ* = 70° which exceeded the critical angle. As the incident angle exceeds the critical angle, the transmittance is vanished and the extinctance *E* defined as the sum of scatterance and absorptance can be derived from reflectance *R* via the relationship *E* = 1 − *R*.

**Figure 4:**
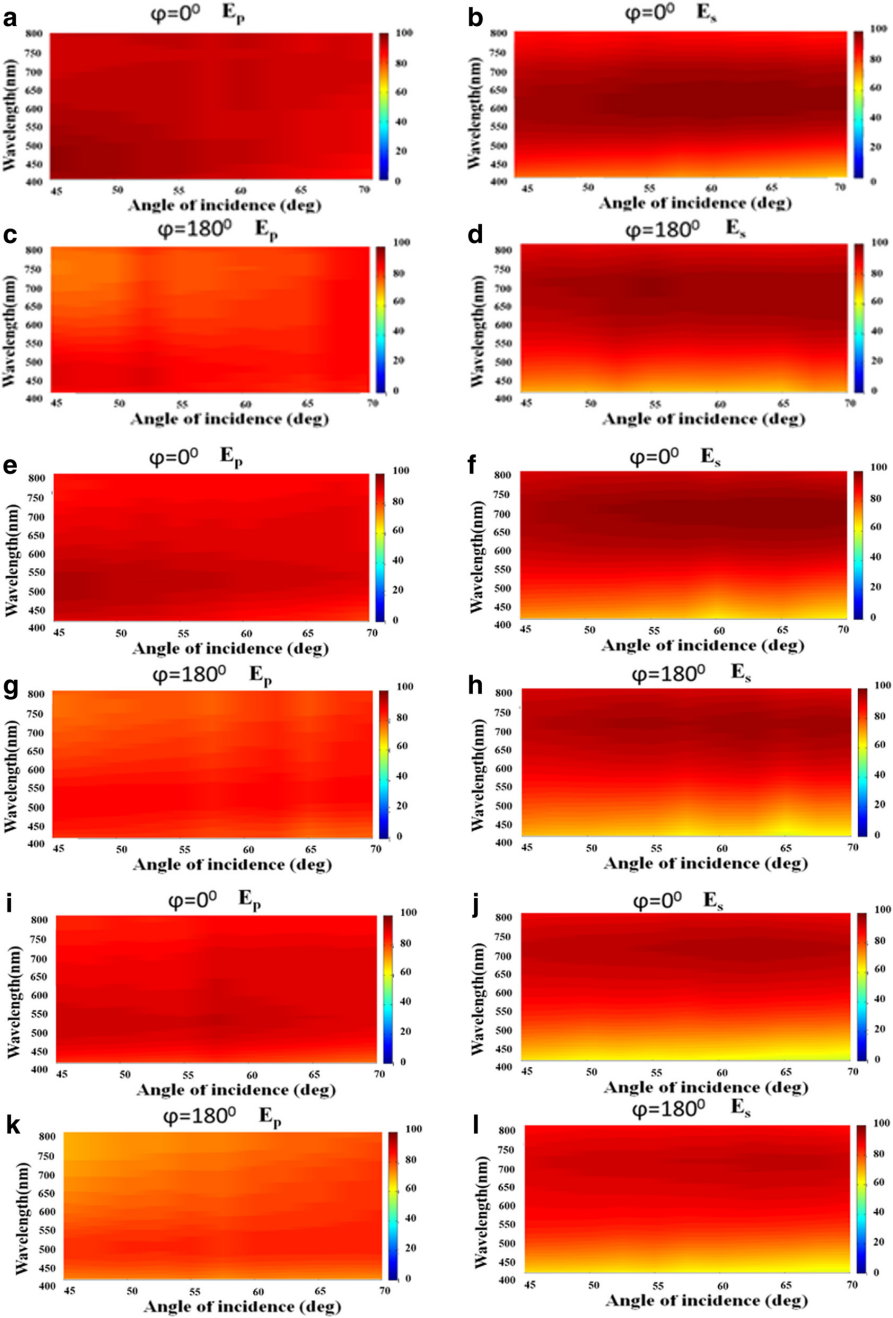
**S- and p-polarized extinctance versus wavelength and angle of incidence.** s-polarized and p-polarized extinctance versus wavelength *λ* ∈ [400 nm, 800 nm] and angle of incidence *θ* ∈ [45°, 70°] for Ag NRAs with thicknesses of **(a, b, c, d)** 169 nm, **(e, f, g, h)** 198 nm, and **(i, j, k, l)** 269 nm.

Figure 4a,b,c,d,e,f,g,h,i,j,k,l plot extinctance *E*_*j*_(*λ*, *θ*) versus wavelength from 400 to 800 nm and incident angle from *θ* = 45° to *θ* = 70° when the Ag NRA was illuminated by *s*(*j* = *s*)-polarized or *p*(*j* = *p*)-polarized light. For the 169-nm-thick NRA that was oriented at *φ* = 0°, the extinctance *E*_*p*_(*λ*, *θ*) and *E*_*s*_(*λ*, *θ*) exceeded 84 and 65% over all of the measured wavelengths and angles of incidence, respectively. In Figure 4a, *E*_*p*_(*λ*, *θ*) exceeds 90% over all of the considered wavelengths at angles of less than 60°. *E*_*s*_(*λ*, *θ*) becomes less than 85% at wavelengths between 400 and 450 nm. The strong *E*_*s*_(*λ*, *θ*) of over 90% is distributed over all angles at wavelengths from 512 to 730 nm, as shown in Figure 4b. When the 169-nm-thick NRA is oriented at *φ* = 180°, the extinctance *E*_*p*_(*λ*, *θ*) is less than that measured at *φ* = 0°, as shown in Figure 4c. A comparison of Figure 4b,d reveals that *E*_*s*_(*λ*, *θ*) that is measured at *φ* = 0° is very similar to that at *φ* = 180° because the growth directions of the rods are vertical to the oscillating direction of the electric field of s-polarization in both cases.

For the 198-nm-thick NRA that was oriented at *φ* = 0° or *φ* = 180°, the extinctance *E*_*p*_(*λ*, *θ*) and *E*_*s*_(*λ*, *θ*) varied with wavelength and angle in a manner similar to the corresponding variations of the 169-nm-thick NRA. The values of *E*_*p*_(*λ*, *θ*) and *E*_*s*_(*λ*, *θ*) in Figure 4e,g are on average 5 and 3% less than those in Figure 4a,c, respectively. The strong *E*_*p*_(*λ*, *θ*) of over 90% is distributed over wavelengths from 508 to 550 nm and angles from *θ* = 45° to *θ* = 70°, as shown in Figure 4e. The values of extinctance *E*_*s*_(*λ*, *θ*) in Figure 4f,h are on average 6 and 3% less than those in Figure 4b,d, respectively. The strong *E*_*s*_(*λ*, *θ*) of over 90% is found at wavelengths from 560 to 800 nm and at angles from *θ* = 45° to *θ* = 70°, as shown in Figure 4f. The difference between *E*_*s*_(*λ*, *θ*) that is measured at *φ* = 0° and *E*_*s*_(*λ*, *θ*) that is measured at *φ* = 180° is only 3%.

The extinctance *E*_*s*_(*λ*, *θ*) and *E*_*s*_(*λ*, *θ*) of the 269-nm-thick NRA that is oriented at *φ* = 0° or *φ* = 180° varies with wavelength and angle similarly to the variations of the other two samples. The values in Figure 4i,k are on average 3 and 5% less than those in Figures 4e,g, respectively. The extinctance *E*_*s*_(*λ*, *θ*) in Figure 4j,l is an average of 5 and 2% less than those in Figure 4f,h, respectively. The difference between *E*_*s*_(*λ*, *θ*) that is measured at *φ* = 0° and *E*_*s*_(*λ*, *θ*) that is measured at *φ* = 180° is only 3%.

**Figure 5:**
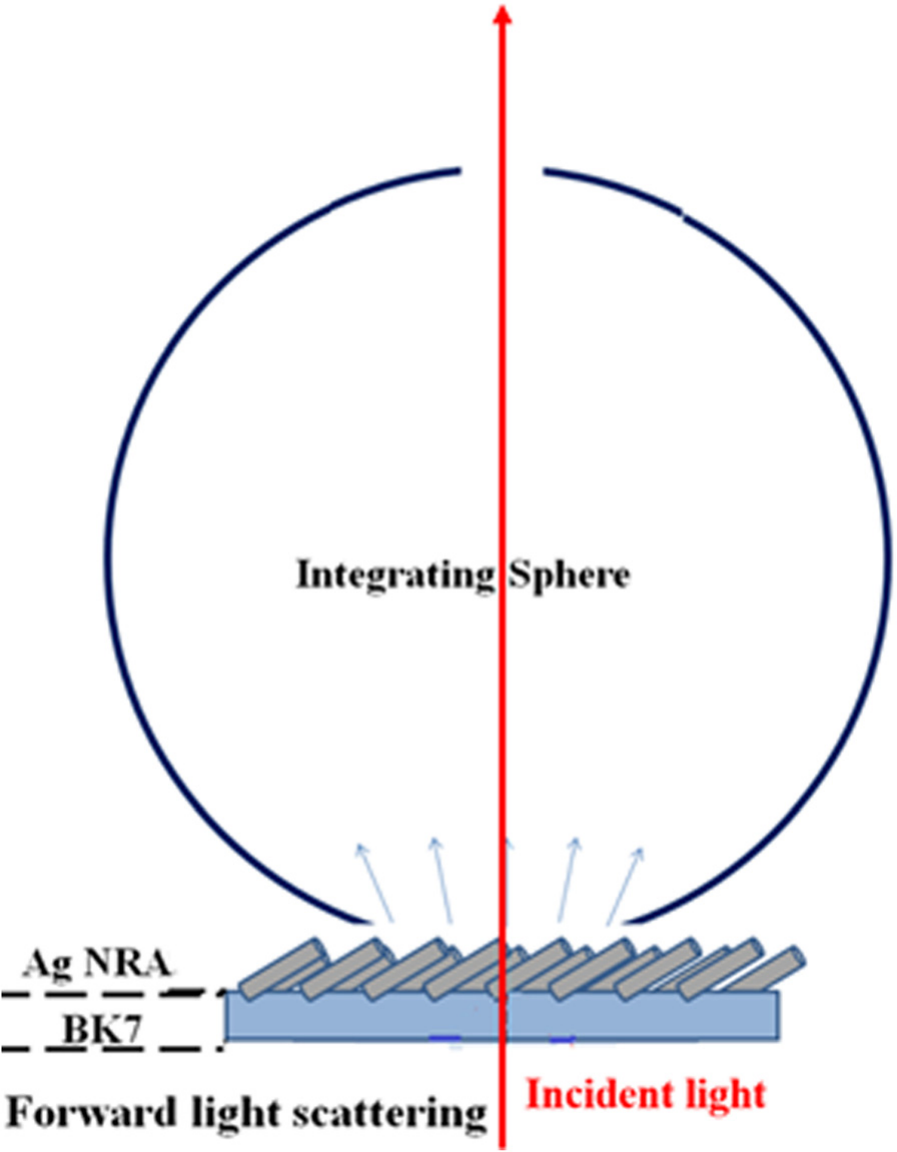
**Scheme of forward scattering measurement for**$S_{{}_{\mathrm{normal}-j}}$ ***j *** **∈ { *p *, *s*} of NRA measured using integrating sphere.**

To investigate the scattering energy from the Ag NRA, an integrating sphere with a diameter of 5 cm is used to measure the forward light scattering. The scattering intensities of the three samples are compared when each is arranged in an air/NRA/BK7 glass system to be illuminated at normal incidence or is arranged in the Kretschmann configuration to be illuminated at oblique incidence. First, the Ag NRA on BK7 glass substrate is illuminated at normal incidence by an He-Ne laser with a wavelength of 632.8 nm. As shown in Figure 5, the forward scattering was measured using the integrating sphere with two openings to prevent the specular beam from interrupting the scattering measurement. As presented in Table 1, the forward scattering intensity under p-polarized illumination is less than that under s-polarized illumination.

**Table 1:** **Scattering intensity**$S_{{}_{\mathrm{normal}-j}}$ ***j *** **∈ { *p *, *s*}, of NRA, measured using integrating sphere for forward scattering**

**Forward scattering**	**169-nm thick**	**198-nm thick**	**269-nm thick**
*S*_normal*-p*_	3.5 (a.u.)	3.7 (a.u.)	5.5 (a.u.)
*S*_normal*-s*_	5.0 (a.u.)	6.8 (a.u.)	12.1 (a.u.)

**Figure 6:**
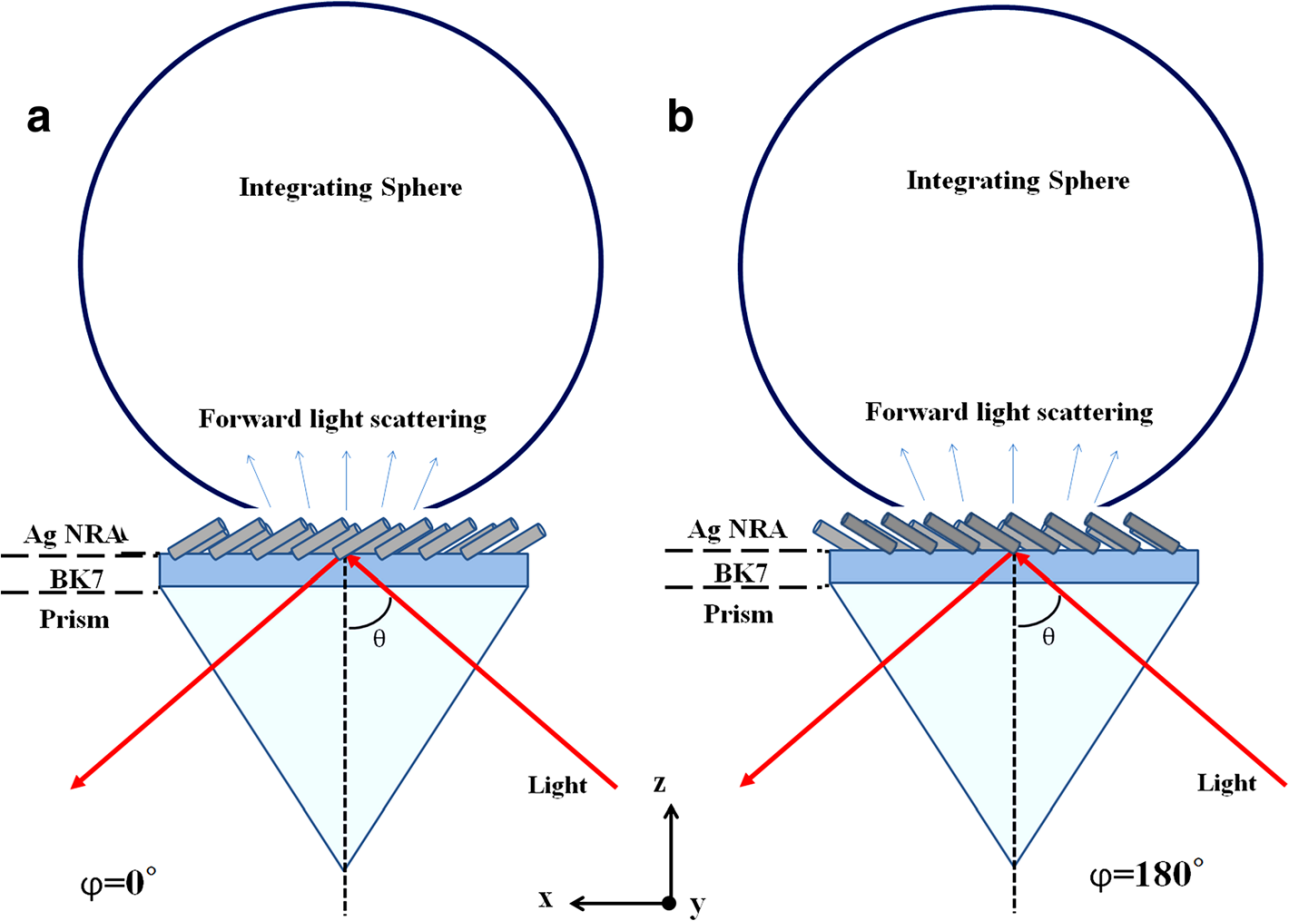
**Scheme of scattering measurement for**$S_{{}_{kr-j}}$***j *∈ {*p*, *s*} of NRA.** This is measured using integrating sphere at orientations **(a)***φ* = 0° and **(b)***φ* = 180°.

**Figure 7:**
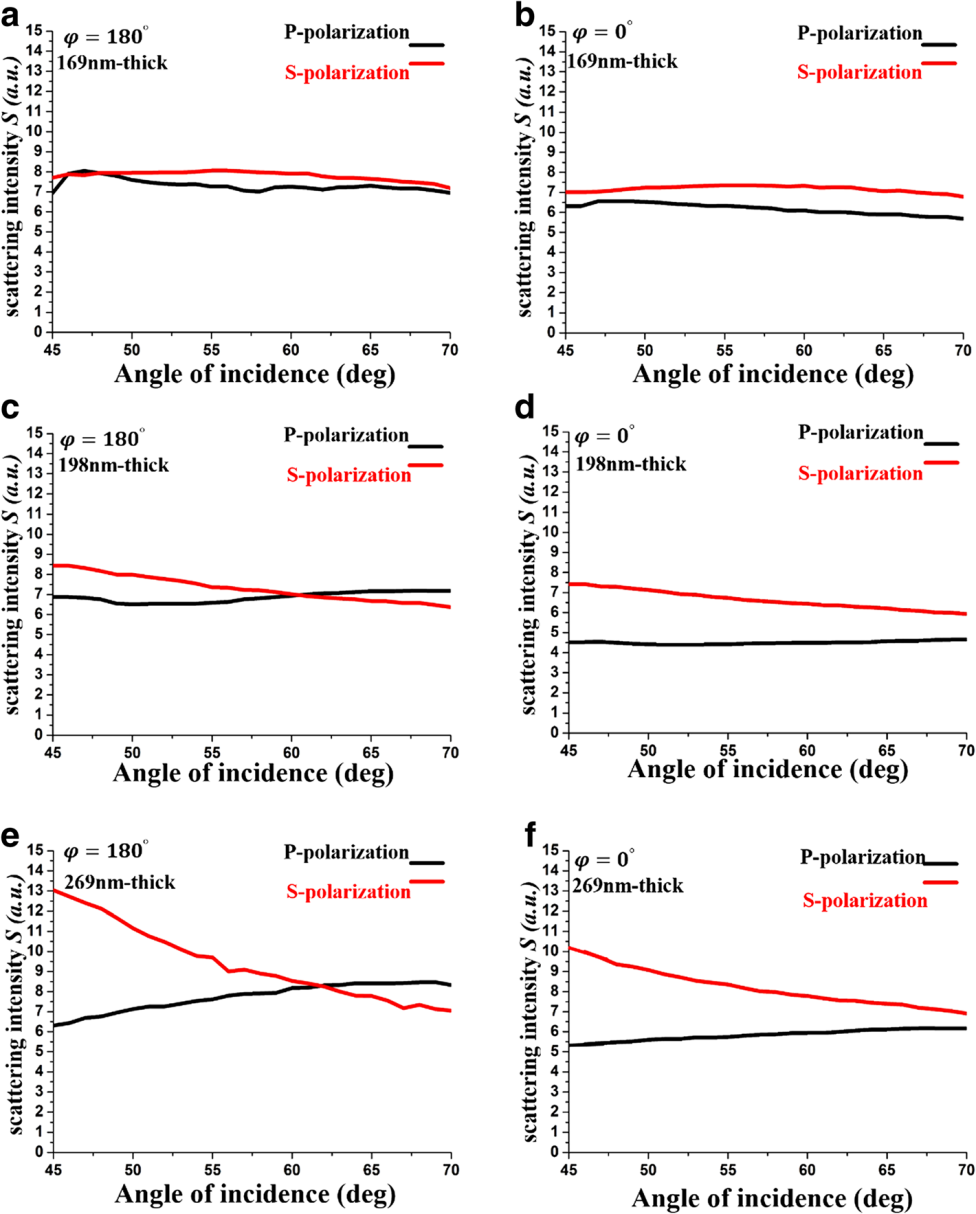
**Measured spectra of**$S_{{}_{kr-j}}$***j *∈ { *p *, *s*} with thickness of 169, 198, and 269 nm.** These are illuminated by differently polarized plane waves with wavelength of 632.8 nm at different orientations. Thickness of 169 nm oriented at **(a)***φ* = 180° and **(b)***φ* = 0°, 198 nm at **(c)***φ* = 180° and **(d)***φ* = 0°, and 269 nm at **(e)***φ* = 180° and **(f)***φ* = 0°.

Next, the Ag NRA is arranged in the Kretschmann configuration and its scattering intensity measured. As shown in Figure 6, the illuminated area of the film falls entirely within the opening of the integrating sphere. As the incident angle is varied from *θ* = 45° to *θ* = 70°, the angular spectrum of the scattering intensity ratio is measured at *φ* = 0° and *φ* = 180° and is in Figure 7. For the 169-nm-thick Ag NRA that is oriented at *φ* = 180°, the scattering intensity *S*_*kr*_ varies from *S*_*kr*_ = 6.928 at *θ* = 45° to *S*_*kr*_ = 6.950 at *θ* = 70° under p-polarized illumination and from *S*_*kr*_ = 7.704 at *θ* = 45° to *S*_*kr*_ = 7.187 at *θ* = 70° under s-polarized illumination. At *φ* = 0°, the measured scattering intensity is approximately 6.165 and 7.176 under p-polarized illumination and s-polarized illumination, respectively. These scattering intensities in the Kretschmann configuration are larger than those under normal illumination because the coupling effect is sufficiently strong that almost 90% of the incident light couples into the rods.

For the 198-nm-thick Ag NRA that is oriented at *φ* =180°, the scattering intensity *S*_*kr*_ is approximately 6.863 under p-polarized illumination and decays from *S*_*kr*_ = 8.696 at *θ* = 45° to *S*_*kr*_ = 6.368 at *θ* = 70° under s-polarized illumination. At *φ* = 0°, the scattering intensity *S*_*kr*_ is approximately 4.501 under p-polarized illumination and decays from *S*_*kr*_ = 7.417 at *θ* = 45° to *S*_*kr*_ = 5.934 at *θ* = 70° under s-polarized illumination.

For the 269-nm-thick Ag NRA that is oriented at *φ* =180°, the scattering intensity *S*_*kr*_ varies from *S*_*kr*_ = 5.592 at *θ* = 45° to *S*_*kr*_ = 8.329 at *θ* = 70° under p-polarized illumination and from *S*_*kr*_ = 13.340 at *θ* = 45° to *S*_*kr*_ = 7.047 at *θ* = 70° under s-polarized illumination. At *φ* = 0°, the scattering intensity *S*_*kr*_ varies from *S*_*kr*_ = 5.284 at *θ* = 45° to *S*_*kr*_ =6.159 at *θ* = 70° under p-polarized illumination and from *S*_*kr*_ = 9.927 at *θ* = 45° to *S*_*kr*_ = 6.908 at *θ* = 70° under s-polarized illumination. The scattering intensities for both polarization states are less than those under normal illumination, although in both cases, the extinctances are similar, at approximately 80%.

The scattering intensity that is measured at *φ* = 180° exceeds that measured at *φ* = 0°. The scattering intensity increases with the thickness of the NRA for both polarization states, but the increase in the Kretschmann configuration is not as high as that under normal illumination.

The broadband and wide-angle extinctance spectra require further measurement and investigation in the future. The equivalent optical constants at normal incidence were described by complex refractive index and complex impedance; both parameters were derived from measured transmission coefficient and reflection coefficient of air/NRA/substrate system. However, the equivalent optical constants at oblique incidence are still under development; the walk-off interferometer for measuring reflection and transmission coefficients needs to be modified. Based on the refractive index and impedance measured at different angles of incidence, the anisotropic property of the metamaterial thin film will be understood, and the mechanism for the strong extinctance in this paper can be revealed.

## Conclusions

When a silver NRA with a thickness of 169, 198, or 269 nm is arranged in the Kretschmann configuration, the broadband and wide-angle extinctions cause strong energy coupling from the incident light to the NRA. High extinction occurs for both s- and p-polarization. The situation differs from that when the same NRA is illuminated normally, when the extinctance is proportional the thickness of the NRA. The highest extinction in the Kretschmann configuration occurs that of the thinnest NRA, with a thickness of 169 nm. Owing to the strong light coupling effect, the 169-nm-thick NRA exhibits stronger forward light scattering in the Kretschmann configuration than that in the air/NRA/BK7 glass system under normal illumination. Since the glancing-deposited silver NRA has been demonstrated to be a highly sensitive and a substrate for surface-enhanced Raman scattering can be easily fabricated, this technique for confining energy within a nanostructure will increase signal strength and sensitivity in bio-sensing.

## Abbreviations

LPM: longitudinal plasmon mode; NRA: nanorod array; SERS: surface-enhanced Raman scattering; TPM: transverse plasmon mode.

## Competing interests

The authors declare that they have no competing interests.

## Authors' contributions

YJJ conceived the idea and supervised the experiment and data analysis. WCL and JHC constructed the optical setup and analyzed the data. WCL, JWH, and YTC fabricated and measured the samples. All authors read and approved the final manuscript.

## References

[B1] Dobrowolski JA Daniel P Penghui M Himanshu V Michael A Toward perfect antireflection coatings: numerical investigation Applied Optics 2002 41 16 3075 3083 10.1364/AO.41.003075 12064383

[B2] Xi JQ Schubert MF Kim JK Schubert EF Chen M Lin SY Liu W Smart JA Optical thin-film materials with low refractive index for broadband elimination of Fresnel reflection Nature Photonics 2007 1 176 179

[B3] Hodgkinson I Cloughley S Wu QH Kassam S Anisotropic scatter patterns and anomalous birefringence of obliquely deposited cerium oxide films Applied Optics 1996 35 28 5563 5568 10.1364/AO.35.005563 21127558

[B4] Jen Y-J Suzuki M Wang Y-H Lin M-J Near-field simulation of obliquely deposited surface-enhanced Raman scattering substrates J Appl Phys 2012 112 113111 10.1063/1.4769806

[B5] Fu J-X Collins A Zhao Y-P Optical properties and biosensor application of ultrathin silver films prepared by oblique angle deposition J Phys Chem C 2008 112 43 16784 16791 10.1021/jp802909g

[B6] Ju J Byeon E Han YA Kim SM Fabrication of a substrate for Ag-nanorod metal-enhanced fluorescence using the oblique angle deposition process Micro & Nano Letters 2013 8 7 370 373 10.1049/mnl.2013.0030

[B7] Mori T Kobayashi T Kawanishi Y Kominami H Nakanishi Y Hara K Fabrication of AlN single crystal particles by a chemical vapor method using aluminum chloride Phys Status Solidi C 2011 8 5 1459 1462 10.1002/pssc.201001115

[B8] Sönnichsen C Geier S Hecker NE von Plessen G Feldmann J Spectroscopy of single metallic nanoparticles using total internal reflection microscopy Appl Phys Lett 2000 77 2949 10.1063/1.1323553

[B9] Jen Y-J Chih-Hui C Ching-Wei Y Deposited metamaterial thin film with negative refractive index and permeability in the visible regime Optics Letters 2011 36 6 1014 1016 10.1364/OL.36.001014 21403760

[B10] Zhao Y-P Chaney SB Zhang Z-Y Absorbance spectra of aligned Ag nanorod arrays prepared by oblique angle deposition J Appl Phys 2006 100 063527 10.1063/1.2349549

[B11] Robbie K Sit JC Brett MJ Advanced techniques for glancing angle deposition J Vac Sci Technol B 1998 16 1115 1122 10.1116/1.590019

[B12] van Kranenburg H Lodder C Tailoring growth and local composition by oblique-incidence deposition: a review and new experimental data Mater Sci Eng R Rep 1994 Rll 295 354

